# Multifunctional Finishing of Cotton Fabric with Curcumin Derivatives Coatings Obtained by Sol–Gel Method

**DOI:** 10.3390/gels9050369

**Published:** 2023-04-30

**Authors:** Florentina Monica Raduly, Valentin Rădițoiu, Alina Rădițoiu, Adriana Nicoleta Frone, Cristian Andi Nicolae, Iuliana Răut, Mariana Constantin, Maria Grapin

**Affiliations:** 1 Laboratory of Functional Dyes and Related Materials, National Research and Development Institute for Chemistry and Petrochemistry—ICECHIM, 202 Splaiul Independentei, 6th District, 060021 Bucharest, Romania; 2 Faculty of Pharmacy, Titu Maiorescu University, Bd. Gh. Sincai, No. 16, 040441 Bucharest, Romania

**Keywords:** curcumin derivatives, sol–gel, hybrid materials, fluorescence, hydrophobic, cotton fabric

## Abstract

Textile materials with fluorescent, repellent, or antimicrobial properties are increasingly used in common applications. Obtaining multi-functional coatings is of wide interest, especially for applications related to signaling or to the medical field. In order to increase the performance (color properties, fluorescence lifetime, self-cleaning or antimicrobial properties) of textile materials with special uses, a series of research was carried out regarding the modification of surfaces with nanosols. In this study, coatings with multiple properties were obtained by depositing nanosols on cotton fabrics generated through sol–gel reactions. These multifunctional coatings are hybrid materials in which the host matrix is generated using tetraethylorthosilicate (TEOS) and network modifying organosilanes:dimethoxydimethylsilane (DMDMS) or dimethoxydiphenylsilane (DMDPS) in a 1:1 mass ratio. Two curcumin derivatives were embedded in siloxane matrices, a yellow one (CY) that is identical to bis-demethoxycurcumin (one of the natural constituents in turmeric) and a red dye (CR) that has a N,N-dimethylamino group grafted in position 4 of the dicinnamoylmethane skeleton of curcumin. The nanocomposites obtained by embedding curcumin derivatives in siloxane matrices were deposited on cotton fabric and studied in relation to the dye and the type of host matrix. Fabrics coated with such systems provide a hydrophobic surface, have fluorescent and antimicrobial properties, change color depending on the pH, and therefore can be used in various fields where textiles provide signaling properties, self-cleaning, or antibacterial protection. The coated fabrics maintained their good multifunctional properties even after several washing cycles.

## 1. Introduction

Currently, there is an increased interest in the development of functionalized textile materials that have new and diverse properties with minimum changes in the classic qualities of maintaining comfort and mechanical resistance. For this, the aim was the development of economic and ecological supports, coverage compositions, and procedures to apply them to textiles at low temperatures without the use of electrolytes, multiple rinsing processes, or fixing agents [1,2,3,4,5]. From the multitude of textile materials modified by various techniques, fabrics with fluorescent properties have been the subject of study for several groups of researchers [6,7,8,9]. These are used in signaling applications (road equipment, sports equipment, materials for special services, advertising, shows, etc.) and they are colored with fluorescent dyes that determine bright and highly saturated hues. In order to increase the performance (color properties, fluorescence lifetime and yield, and resistance to photodegradation) of materials with special uses, a series of research was carried out regarding the modification of the surfaces with nanosols. Finishing textile fabrics made of natural or synthetic fibers with various compositions of nanosols have led to flexible coatings with repellent, flame retardant, antimicrobial, self-cleaning, and UV protection properties [9,10,11,12,13,14]. The development of procedures for the ecological dyeing of textile fibers has brought natural dyes to the attention of researchers [15,16,17,18], which are biodegradable, do not produce allergies, and some of them show antioxidant and antibacterial properties. However, there are some shortcomings regarding the use of natural dyes for dyeing textile materials, especially due to their weak light resistance. Therefore, in order to compensate for this deficiency, dyeing is performed simultaneously with mordants, resins, tensides, nanosols, or after pretreatment of the fibers, depending on the nature of the fabric [9,18,19]. These treatments increase the light resistance of the dyes, but unfortunately, in the case of structures such as curcumin derivatives, the formation of metal-complexes leads to the quenching of fluorescence [20]. It should be mentioned that the signaling equipment is obtained only by coloring in shades of yellow, orange, and red, usually having and imparting fluorescence to the textile support. Given that fluorescent dyes belong to a small number of coloring classes based on the affinity for the fibers (e.g., cationic or dispersion dyes) and pigments are insoluble species, the situation of limiting the application procedures on textile supports becomes evident [21,22]. The mass coloring process is widely spread and intensively used for all types of signaling textiles made of polyesters. The problems related to this process refer to obtaining limited shades, the short lifetime of fluorescence, the quenching of fluorescence through interactions with the support and with other dye molecules, and the poor resistance to photodegradation, finally leading to the need to replace the signaling material.

Moreover, the opening of science to the general public aims to educate humanity on a large scale regarding hygiene and methods of increasing the levels of health. In this sense, several studies have been published on nosocomial infections and methods of limiting their transmission [23,24,25,26]. The most exposed methods of the transmission of bacteria and fungi are the textile materials used in hospitals, for example, protective uniforms or bed linen. Thus, textile-finishing methods have been studied using different agents with biocidal activity such as metal or metal oxide nanoparticles, quaternary ammonium compounds, triclosan, dyes, and chitosan to obtain antimicrobial textiles [27,28,29,30,31]. Depending on their nature, these biocidal substances have been conditioned in the form of nanoparticles, nanocomposites, and gels, and then encapsulated in organic or inorganic matrices to favor their most efficient application as a finishing agent or incorporated into the fiber without losing their initial properties [32,33,34,35,36,37,38]. One of the most widely used finishing methods for textile fabrics is to deposit nanosols obtained by the sol–gel method. This is easy to apply and very versatile in terms of the active compounds that can be hosted in the resulting gels. Coatings of this type lead to fabrics having various antimicrobial, UV protection, or water repellent properties [10,14,32,39].

In the present paper, we propose a study on obtaining hybrid materials with multifunctional properties determined by the structure of the dye embedded in the siloxane matrices prepared by the sol–gel method. The functionalization of cotton fabric surfaces with nanosols in which curcuminoid dyes with different auxocromes (Supplementary Materials, Figure S1, Table S1), hydroxy (CY), or N,N-dimethylamino (CR) are embedded, have resulted, obtaining textiles with the following properties: fluorescence (yellow—CY1, CY2 or red—CR1, CR2), antimicrobial, and hydrophobic. Following the finishing of textiles with hybrid materials obtained through the sol–gel process, the development of the range of natural fabrics with higher quality properties compared to the natural fabrics dyed by classical methods [18,19] is targeted.

## 2. Results and Discussion

**Figure 1:**
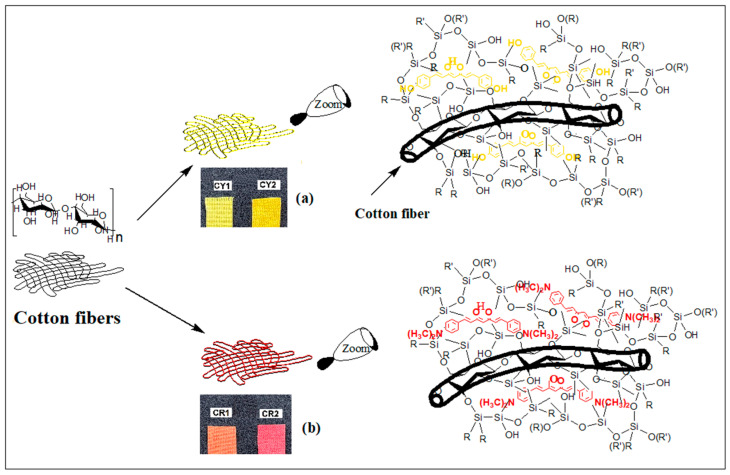
Cotton materials coated with yellow CY1, CY2 (**a**) and red CR1, CR2 (**b**) colored nanocomposites (R: CH_3_, C_6_H_5_; R’: H, C_2_H_5_).

In order to study the behavior of film-forming materials in which two types of curcumin derivatives were incorporated through the sol–gel process, four types of nanocomposites with different silica network modifiers were synthesized. The hybrid materials obtained were deposited on a cotton textile support and studied from a morphological and structural point of view. In this way, two yellow cotton samples were obtained (Figure 1a) that were uniformly covered after the impregnation process with nanosol solutions containing bis-demethoxycurcumin (identical to the natural dye) sequestered in the silica host matrices modified with aliphatic (CY1) or aromatic (CY2) residues. Using the same method, two red cotton samples were obtained (Figure 1b) after impregnation with nanosols containing a curcumin derivative with N,N-dimethylamino groups instead of hydroxy groups (synthetic dye) sequestered in the silica host matrices modified with aliphatic (CR1) or aromatic (CR2) residues. Depending on the type of dye and the nanocomposites deposited on the fabrics, the fluorescent, antimicrobial, and water repellent properties of the four types of hybrid materials were studied.

### 2.1. Structure and Morphology of the Coated Fabrics

**Figure 2:**
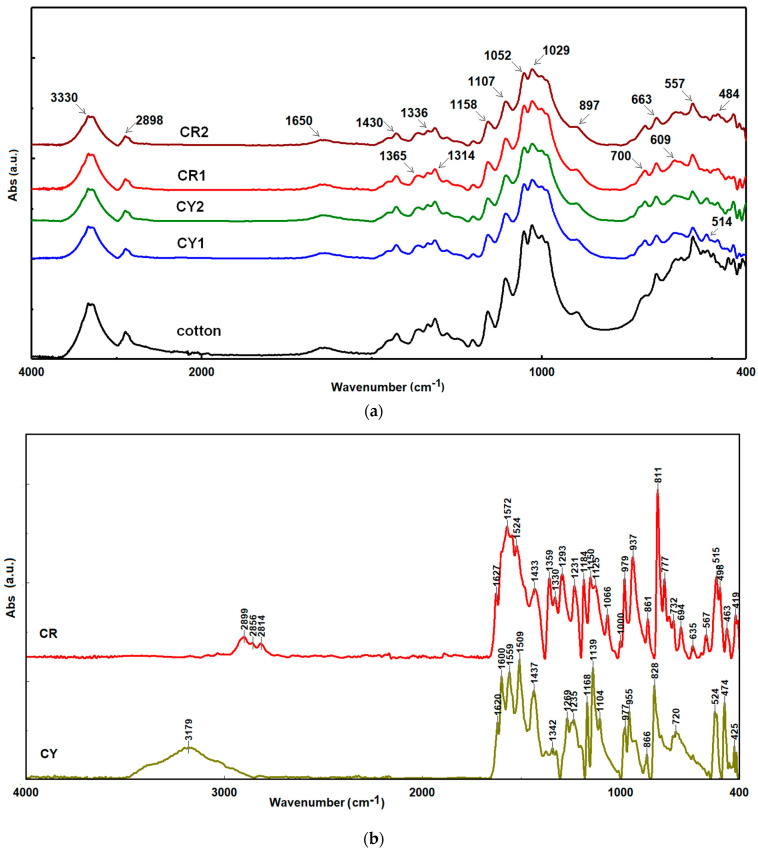
FTIR-ATR spectra for raw and cotton fibers coated with nanosols (**a**) and curcumin derivatives (**b**).

The analysis of the FTIR-ATR spectra of the fabrics covered with nanosols and the cotton support (Figure 2a) showed many similarities, and at the same time, many of them overlapped the vibration bands characteristic of curcumin derivatives (Figure 2b). It was the case of broad O–H stretching bands at 3330 cm^−1^ that overlapped with the bands at 3179 cm^−1^ attributed to O–H from CY, while the region 2890–2850 cm^−1^ was assigned to C–H stretching vibrations and were found in both the textile samples and in curcumin derivatives. Next, a broad vibration band at 1650 cm^−1^ attributed to the O–H bond of adsorbed water was observed. This band overlapped with the vibration band at 1620 cm^−1^ characteristic of C=C stretching of the α,β-unsaturated ketone, and the area in the range of 1430–1300 cm^−1^ showed bands characteristic of the scissoring and wagging vibrations of methylene groups, which were found in both the structure of the dyes and cotton fibers (Figure 2b).

The bands at 1158, 1107, 1052, and 1029 cm^−1^ were attributed to the stretching vibrations of the C–O bonds found in the structure of the textile support [40,41,42] and curcumin derivatives. Simultaneously, they can also be attributed to the Si–O bonds in the silica matrix of the nanosols, so the two areas overlapped and made their selective identification impossible. This represents a disadvantage for the identification and characterization of nanocomposites, so that more attention was given to the range situated under 900 cm^−1^, in which the cotton support had a reduced number of characteristic signals. In this region, only a characteristic β-glycosidic linkage band was seen, which was present at 897 cm^−1^, and the band at 663 cm^−1^, which was attributed to the O–H out-of-plane bending. At the same time, at 700 cm^−1^, a vibration band characteristic of aromatic rings was observed and attributed to curcuminoid derivatives and phenyl residues from the silica host matrix (type 2). The broad bands at 516, 484, and 450 cm^−1^ were attributed to Si–O asymmetric stretching vibrations, respectively, to O–Si–O, similar to the bands recorded for natural silicates [43,44].

The morphology of the silicon network obtained through the gelation process is important due to the internal interactions that can be established between the host matrix and the guest curcumin derivative, and also by the external interactions that the nanocomposite network establishes with the textile support. From the analysis of the absorption–desorption isotherms of the coated textile materials, it was observed that the nanocomposites whose silica network was modified with aliphatic groups formed coatings with a compact structure that joined the meshes between the threads of the fabric, and the S_BET_ surface was reduced (Table 1) compared to the raw material surface. At the same time, it was observed that the chemical structure of the curcumin derivative influenced the morphology of the coatings obtained with the same type of host matrix. Thus, the hydrophobic character of the CR structure, given by the auxochrome groups, led to the establishment of van der Waals or hydrogen intermolecular bonds as well as the π−π stacking between the aromatic rings of the dye and the host matrix, and generates structures with a smaller volume, in comparison with the nanocomposites accommodating the CY dye. The auxochrome hydroxyl groups on the aromatic rings of the CY dye structure established hydrogen bonds with the host matrix, leading to nanosols with a larger total volume and pore diameter than those containing the CR dye. The replacement of methyl-type network modifying groups with phenyl groups generated silica networks with a much larger surface and volume, modifying the architecture of the host matrix, which will directly influence the properties of the dyes through the tautomeric structures they can establish in the siloxane matrix.

**Figure 3:**
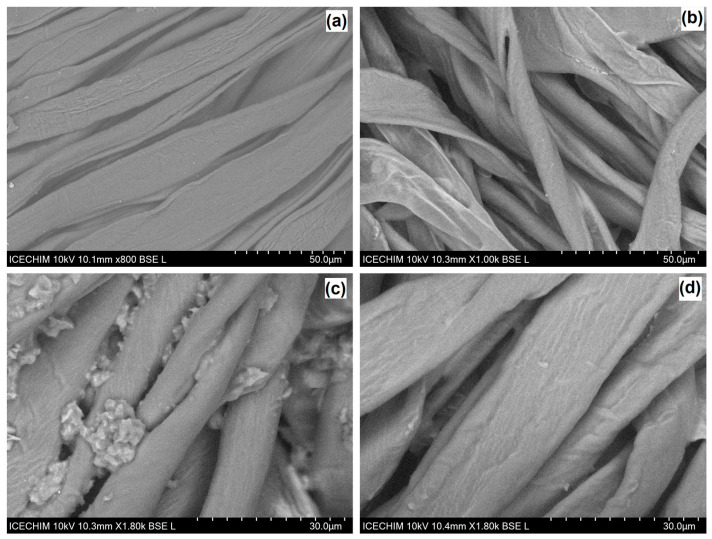
SEM micrographs of the raw (**a**) and coated cotton fabrics: CY1; (**b**) mass ratio TEOS:DMDMS 1:1, CY1; (**c**) mass ratio TEOS:DMDMS 1:1, +50% dye, CR22; (**d**) mass ratio TEOS:DMDPS 1:2.

By comparing the SEM images of the uncoated fabric (Figure 3a) and those of the coated materials, it can be seen that the morphology of the dyed fabrics was influenced by the composition of the nanosols, especially by the mass ratio between the dye and the host matrix. A mass ratio of 1:1 between TEOS and the network modifiers, which generate the host matrix, determine a uniform coating on the fabric, as can be seen in Figure 3b. A 50% increase in the proportion of the modifier in the siloxane network or rising the amount of curcuminoid dye can affect the properties of the obtained nanosols. As can be observed from Figure 3c, a larger amount of CY can lead to strong intramolecular bonds and thus the formation of aggregates, and the coatings on the cellulose support were uneven and with low resistance. If the proportion of dye is kept constant and only the structure of the host matrix is modified (e.g., the mass ratio of TEOS:DMDMS or TEOS:DMDPS becomes 1:2), nanosols 12 and 22 are obtained. These types of siloxane matrices that host curcumin derivatives did not generate qualitatively modified coatings on the cotton substrate. With these type of nanosols, uniform coatings were achieved on the fabric, as can be seen in the case of CR22 (Figure 3d).

**Figure 4:**
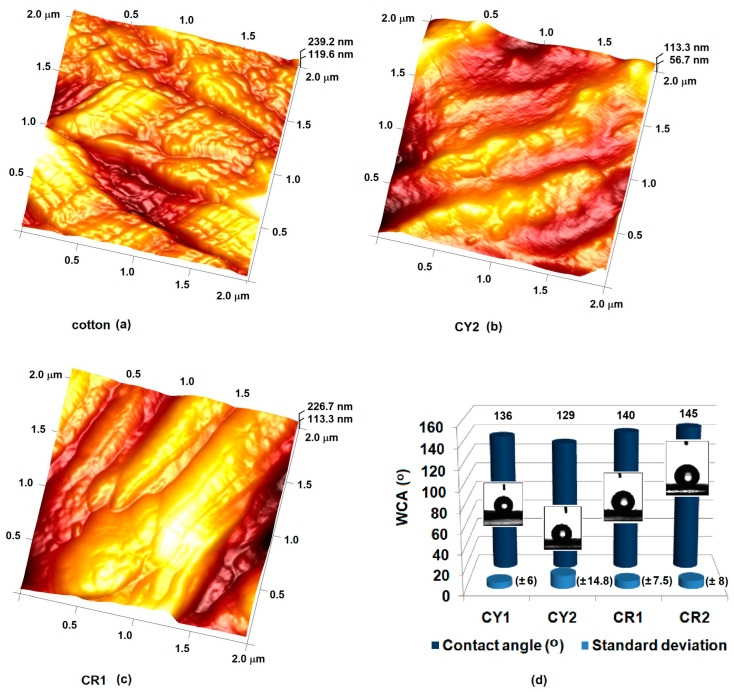
Topography of raw (**a**), hybrid materials CY2 (**b**), CR1 (**c**), and the contact angle of water on coated cotton fabrics (**d**).

The topography of the coatings was analyzed by the AFM method. After mapping the surfaces, it was observed that the nanocomposites with aromatic residues had a peak height around Rmax = 113 nm (Figure 4b), almost half of the peak height recorded for the uncoated fabric (Figure 4a). This was probably due to the π−π stacking of the phenyl groups introduced through the DMDPS network modifier and their orientation toward the surface due to their hydrophobic character. The assumption was also supported by the results of the porosimetry analyses, in which the hybrid materials of type CY2 and CR2 were characterized by large surfaces (S_BET_) and the pore volume (V_tot_), which were almost three times higher compared to CY1, respectively CR1 (Table 1). These coatings were characterized by a higher roughness (square roughness of CY2 R_q_ = 112.37 ± 7.6 nm) compared to the coatings obtained on the same type of hydrophilic support, but in which the network modifiers introduced through DMDMS had a considerably smaller volume (square roughness of CY1 R_q_ = 82.67 ± 4.3 nm). Thus, the coatings obtained from nanocomposites modified with methyl groups had a peak height of approximately Rmax = 227 nm (Figure 4c). The lower roughness of the hybrid materials (square roughness, CR1 R_q_ = 56.4 ± 7.1 nm) obtained from nanosols modified with methyl groups determines contact angles of approximately 136° ± 6 (CY1), respectively 140° ± 7.5 (CR1) measured against water. The increased roughness of the CY2 type coatings led to a decrease in the contact angle to approximately 129° ± 14.8, but in the case of CR2, most likely the N-methyl groups in the aromatic structure of the CR dye caused an increase in the roughness (square roughness of CR2 R_q_ = 57.19 ± 2.6 nm) due to the arrangements π−π between the aromatic rings and the orientation toward the outside of the matrix of the methyl groups implicitly determining the increase in the contact angle to 145° ± 8 (Figure 4d) [45].

**Figure 5:**
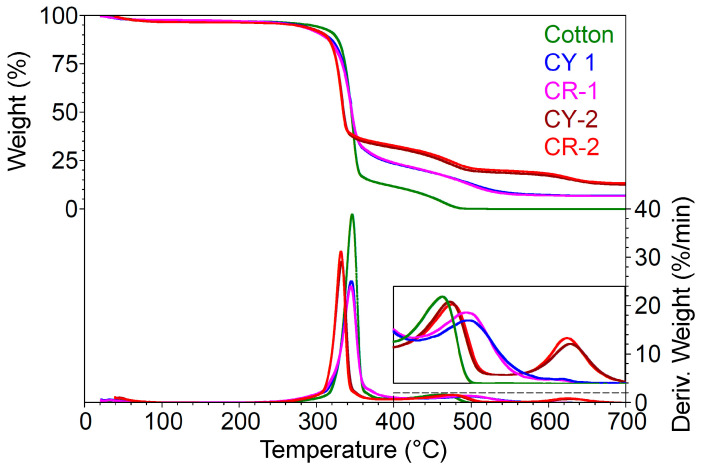
Thermogravimetric curves of the coated cotton with nanosols.

High-resolution thermal gravimetric analysis was applied to the four types of finished textiles and to the raw textile support (Figure 5). A dynamic heating rate was applied to improve the resolution of the last step decay processes within a reasonable measurement time. Under these conditions, it was observed that up to 135 °C for all samples, there were insignificant mass losses of around 3% due to the evaporation of water and residual solvents. The thermal process that takes place with maximum intensity at approximately 300 °C differed for each sample depending on the structure of the silica network. Thus, the samples covered with nanosols modified with phenyl groups had a mass loss of approximately 60% at 304 °C compared to the raw material, which lost 75% of its mass at 313 °C [40]. By comparison, among the nanosols modified with methyl residues, CY1 lost more, probably because of the weaker interactions between the nanosol and the support textile material. The decomposition processes started faster in the dyed samples but they were less intense compared to the burning processes of the uncoated cotton. These thermal phenomena occurred due to the volatile properties of the organic compounds present in the nanosols. The second stage of the thermal decomposition process took place faster for the uncovered fabric (446 °C). Thermal effects lead to the breaking of C–C and C–O bonds, thus resulting in the carbonization of cellulosic fibers with a mass loss of 20%, obtaining a residue of 1.6%. For the coated samples, this stage took place at 10–17 °C higher temperatures and the mass losses were slightly lower. Finally, a decomposition process took place around the temperature of 615 °C. In this stage, which is missing in the decomposition process of the uncovered cotton fabrics, the mass loss was approximately of about 5–6% and the definitive damage to the structure of the silica network probably occurred by breaking the Si–O bonds. At the end of the thermal decomposition process of the coated samples, a residue of 10–13% consisting of inorganic compounds was obtained.

### 2.2. Optical Properties of the Coated Fabrics

To evaluate the optical properties of the hybrid materials, the absorption and diffuse reflection spectra were measured. All nanocomposite coatings hosting curcumin derivatives absorbed around 420 nm (CY1), with a hypsochromic shift of 26 nm in the case of CY2 due to the intermolecular hydrogen bonds between the CY dye and the host matrix. These intermolecular interactions between the host matrix and the guest dye are similar to the bonds formed when the dye is dissolved in polar solvents. As a result, the absorption maximum only differed by 5–10 nm between the two colored systems. In the case of the CR dye, due to the lower electronegativity of the nitrogen compared to the oxygen atom leading to a higher conjugation availability of unpaired electrons with aromatic residues, the transition states were found in the asymmetric appearance of the absorption band, with a shoulder toward 500 nm (Supplementary Materials, Figure S2). The sol–gel materials that hosted the CR derivative had their absorption maximum bathochromically shifted to 440 nm (CR1) and 434 nm for CR2 (Table 2).

The difference between the two was insignificant because the bonds between the N,N-dimethylamino auxochromes of CR and the host matrix were weaker compared to the hydroxyl groups, which were on the structure of the CY derivative that established polar bonds with the siloxane matrix. These hypotheses were also supported by the K/S coefficient values calculated using the Kubelka Munk equation, where K is the absorption coefficient and S is the scattering coefficient of the coated materials. Knowing that the concentration of the dye (CY or CR) is the same, the influence of the matrix can be observed depending on the siloxane network modifiers. As the possibilities, from a structural point of view, to establish polar bonds between the dye and the host matrix are reduced, the values of the K/S coefficient increase (Table 2). At the same time, the intermolecular bonds between the dye and the modifying groups of the siloxane network with a small volume due to the methylene type of modifier and the intermolecular bonds of the hydrogen bond type between the nanosols and the hydroxyl groups on the cellulose fibers constitute a combination of factors that lead to more resistant coatings (Supplementary Materials, Figure S3). This was evidenced by a moderate decrease in the K/S coefficient for the hybrid materials that hosted CY or CR in nanosol type 1 siloxane matrices compared to those of nanosol type 2 where a decrease of 46–50% was observed after 15 repeated washing cycles.

**Figure 6:**
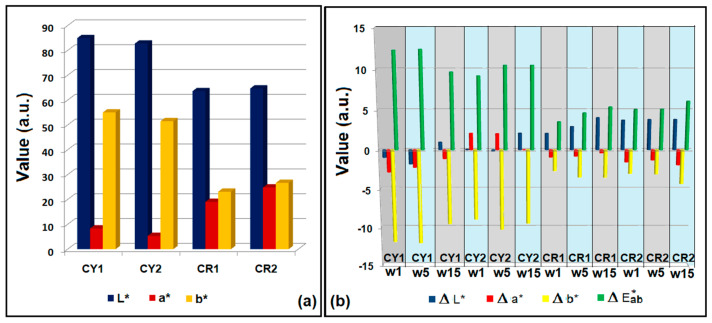
Color parameters in the CIEL*a*b* system from CY1, CY2, CR1, CR2 (**a**) and the evaluation of the films’ resistance during repeated washing cycles (w1–w15) by the measurement of ∆E* in the CIEL*a*b* system (**b**).

**Figure 7:**
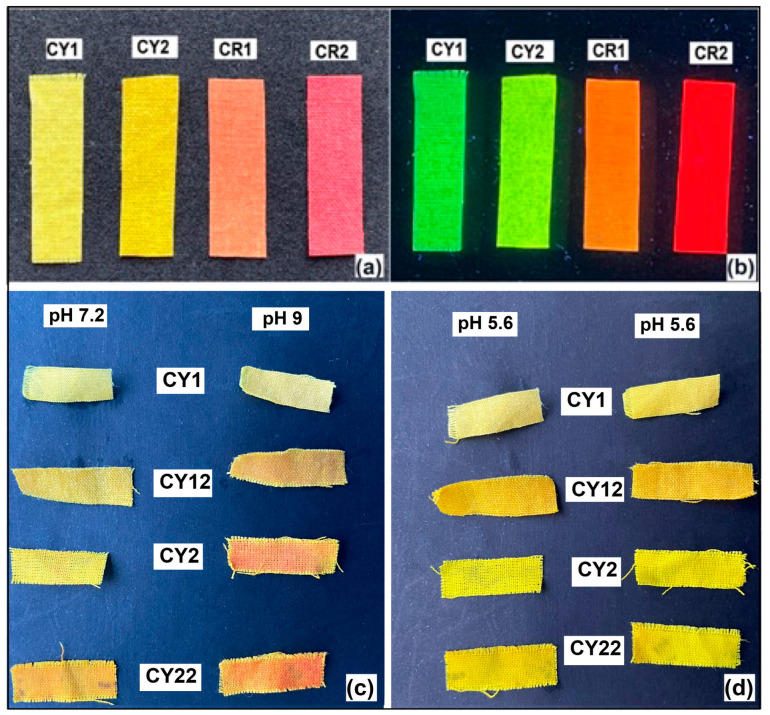
Cotton textiles coated with nanosols (**a**) and the fluorescence emission during excitation at 365 nm (**b**). Color change depending on pH for samples coated with nanosols that hosted the curcuminic derivative CY in alkaline (**c**) and weakly acid (**d**) environments.

The color parameters for the four types of fabrics covered with nanocomposites were measured in the CIEL*a*b* system using a 10° observer and illuminant D65. It can be seen from the analysis of the obtained data that the host matrix modified with methyl groups led to bright hybrid materials with high lightness L* = 86.96 and a shift of the hue toward yellow b* = 55.03 (CY1) as a result of the hydrogen bonds established between the hydroxyl auxochromes and the silica network. In the case of the CR1 hybrid material, the lack of intermolecular polar bonds led to a decrease in lightness L* = 63.61 and the a* parameter increased, the hue being shifted toward red. The introduction of benzene groups into the silica network determines the establishment of π−π type bonds between the dye and the siloxane matrix. These types of intermolecular bonds have the effect of decreasing the brightness in the case of CY2 and closing the shade of CR2 without affecting the brightness by increasing the value of a* = 24.84 (Figure 6a). The optical image of the four coated textile materials are presented in Figure 7a. To evaluate the dyeing performances, the coated fabrics were subjected to a cycle of 15 repeated washing cycles. After each washing cycle, the color parameters of the washed and dried fabric were measured. The total color difference was calculated with Formula (1).

(1) $$∆Eab=\sqrt{∆L^{*2}+∆a^{*2}+{∆b}^{*2}}$$

where L* is the lightness value (0 for black and 100 for white); a* is the position between the red and green value (negative—green, positive—red); b* is the position between the blue and yellow value (negative—blue, positive—yellow).

The results obtained after 15 washing cycles showed no significant changes in ΔE*ab. The samples subjected to the washing process, compared with the unwashed materials, revealed that the coatings with the sol–gel compositions that had the host matrix modified with methyl groups were more resistant (Figure 6b). These results were also supported by comparing the K/S coefficients measured for the washed samples, which showed that for the hybrid materials CY2 and CR2 (Table 2), the coefficient decreased by half compared to the initial one. The influence of the host matrix through its morphology and the types of bonds that it can establish with the hosted dye on the one hand, and with the cotton support on the other, was also observed in the washing fastness and rubbing fastness tests. Table 3 shows the results obtained for the washing fastness and rubbing fastness according to ISO 105 C06 ISO and 105 X12, respectively. These confirm the intermolecular bonds established between nanosols and the cotton fabric through relatively good adhesion performance.

During the washing processes, color changes were observed in the case of the nanocomposites hosting the CY curcumin derivative. Since the color change process was reversible, we considered it necessary to test the coated fabrics at different pH values. The tests were carried out in buffer solutions with pH = 7.2 and pH = 9 by immersing the samples for 10 min. After the exposure time, it was observed that depending on the pH of the solution, the same type of hybrid material changed its color from yellow-orange at pH = 7.2 to orange-red at pH = 9 (Figure 7c). The samples immersed in the alkaline solution were then washed with distilled water (pH = 5.6), and the color returned to the initial yellow shades of immersion in the alkaline buffer solutions (Figure 7d).

In the specialized literature, many results have been presented regarding the properties of curcumin to change its color depending on the pH. These studies implicitly led to the development of curcumin applications as a pH indicator or color sensor [46,47,48]. Thus, the color changes due to the presence of hydroxyl groups in the structure of curcumin were also expected for the curcumin derivative CY with a similar structure. These properties were influenced in the present case by the structure of the siloxane matrix that hosted the dye. Due to the large contact angles of the coated materials, the wetting time of the fabrics was increased. The introduction of network modifiers in a mass ratio of 1:2 compared to TEOS generated siloxane networks with modified structures (CY12), which facilitated the exposure of the CY dye to the alkaline environment. The wetting process favored by the modification of the structure of the siloxane network involves the interactions with the hydroxyl groups in the CY structure and results in the transition of the dye molecule in the anionic form and the coloring of the textile material in orange-red. When the pH changes in an acidic environment, the color of the fabric returns to yellow, a sign that the structure of the curcumin derivative has returned to its neutral, initial form.

The textile samples covered with the organic–inorganic hybrid materials (Figure 7a) showed yellow-green, orange-red fluorescent properties (Figure 7b). All analyzed fabrics showed a fluorescence emission maximum at 470 nm due to the π−π* and n−π* transitions that are characteristic of α,β-unsaturated carbonyl compounds.

**Figure 8:**
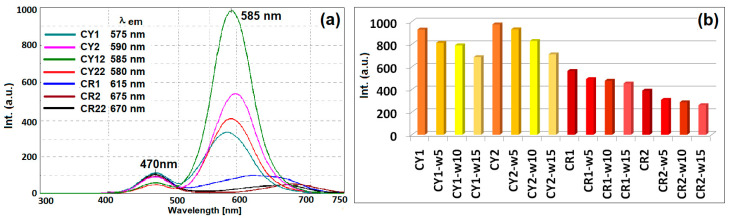
Fluorescence for dyed textiles (**a**) and the decrease in its intensity after fifteen washing cycles (**b**).

Depending on the structure of the inorganic silica network in which curcumin derivatives are embedded, it was observed that the interactions established between the dye and the methyl/phenyl groups in the siloxane network determine the mesomeric structures of the dye that emit fluorescence, with a red shift of the emission wavelength of 55–75 nm (Figure 8a) compared to the curcumin derivative CY in a polar solvent [49,50]. Due to the extension of the conjugation through intermolecular hydrogen bonds established between the hydroxyl auxochromic groups and the host matrix, the displacement of the fluorescence emission maxima was smaller compared to the hybrid materials hosting CR, which shifted at longer wavelengths by about 70–130 nm. At the same time, amine group-type auxochromes had the effect of shifting the emission band to higher wavelengths toward 675 nm, but with a major diminishing of the fluorescence intensity.

If, in general, the fluorescence processes are influenced by the polarity of the solvents in which the dyes are dissolved, in this case, the intensities of the emission effects were influenced by the different proportions of siloxane derivatives that modify the structure of the silica network, and implicitly, the morphology of the obtained gels. In this case, it was observed that an increase of 50% in the mass ratio of DMDMS led to the generation of neighborhoods that considerably improved the fluorescent emission processes of gels containing CY. Increasing the mass ratio of the DMDMS network modifier had the effect of raising the isolation of the dye from the host matrix. These processes have the effect of diluting the concentration of the dye in the vicinity of the surrounding matrix, similar to the dilution processes in non-polar solvents that lead to the increase in the fluorescence intensity. In the case of CY sequestration in the host matrix, the fluorescence quenching processes were influenced, on one hand, by the structure and size of the cages generated in the silica network that determine the percentage of the hosted dye and the intermolecular bonds established between it and the host. On the other hand, the morphology of the host matrix influences the fluorescence processes through the size of the formed pores that determine the evaporation processes of the solvents in the hybrid system, and implicitly, the aggregation processes of the dye molecules.

However, further increasing the proportion of DMDPS in the generation of the silica network has the effect of quenching the fluorescence processes, probably due to the stacking conformations between the benzene nuclei in the host matrix and those of the curcuminoid dye. In the case of gels containing CR with amino groups grafted on the benzene cores, much lower intensities of the emission effects were observed, probably induced by the aggregation effects between dye molecules with lower solubility than CY or the prevention of electronic conjugations due to the non-planar configurations of the molecules of dye. The decrease in the intensity of the fluorescent emission processes after repeated washings was more pronounced in the case of the fabrics coated with nanosols modified with phenyl groups (Figure 8b) and is in agreement with the results of the dyeing performance after repeated washing tests. Thus, the reduction in the intensity of the maximum fluorescence emission by 19–25% after the repeated washing process confirms that the CY1 and CR1 coatings were more resistant compared to the type 2 ones (27–33% reduction in intensity).

**Figure 9:**
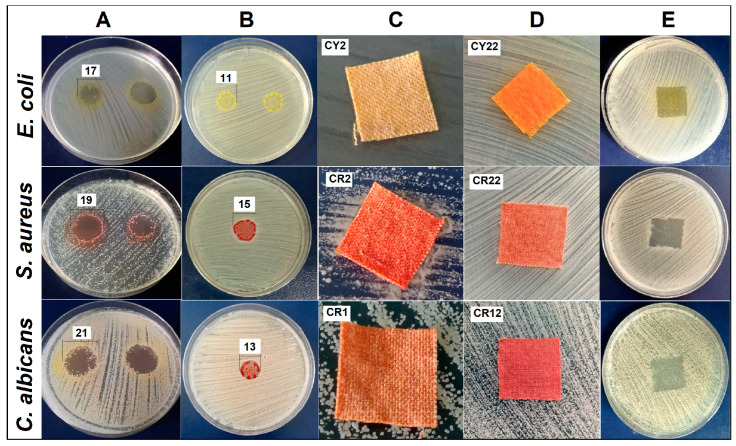
Antimicrobial activity of (**A**) curcumin derivatives and (**B**) sol–gel hybrid materials (numbers in figure represent halo diameter measured in mm); (**C**,**D**) images of textile specimens inoculated on microbial strains and (**E**) the bacteriostatic/fungistatic effect of dyed fabrics.

The fabrics covered with the hybrid materials were tested to evaluate the antimicrobial activity against the fungi *C. albicans* and the bacteria *E. coli* and *S. aureus*, the antimicrobial activity of curcumin being well-known [51,52,53,54]. In order to carry out a more eloquent study, the alcoholic solutions of the two curcumin derivatives (Figure 9A) and the gels obtained following the sol–gel embedding processes of dyes in siloxane matrices (Figure 9B) were initially tested at the same concentration. Then, the fabric samples coated with the respective nanosols were evaluated (Figure 9C–E). Curcumin derivatives tested in an alcoholic solution showed a strong antifungal activity, the average diameter of the halos being measured in the range of 21–23 mm. Antibacterial activity was more intense on cultures of Gram-positive bacteria, where the diameters of the halos were measured in the range of 24–14 mm. After incorporating the curcumin derivatives into the host matrices, the obtained gels showed antibacterial activity. However, these properties were influenced by the nature of the siloxane matrix, by the intermolecular bonds established between the inorganic matrix and the hosted dye. Thus, it was found that the nanosols with CY content considerably lost the antibacterial activity of the dye, especially for fungal strains (approximately 40–60%). This effect was probably caused by the involvement of the hydroxyl groups in the structure of the dye in the establishment of intermolecular bonds with the siloxane matrix, thus leading to a decrease in the biocidal effect. For the gels containing the CR dye, the decrease in antibacterial activity was lower, around 20–30%, and can be justified by the limited access of the curcumin derivative to interact with microorganisms. However, the stratification processes between the benzene rings of the matrix and those in the CR structure led to steric orientations of the dye with auxochromic groups toward hydrophobic environments. In these conditions, cotton fabrics that have hydrophilic properties, after being covered with the nanosols obtained through sol–gel processes, will gain new water repellent properties. These properties help to preserve the antimicrobial activity of the gels and the acquisition of a bactericidal/fungicidal effect of the tissue (Figure 9E).

Similar to the data from the literature regarding the antimicrobial properties of fabrics covered with various film-forming materials [55,56,57], the growth of microorganisms under the textile specimen was evaluated. Textile samples CY1 and CY2 showed no antimicrobial effect, their limited efficacy being evidenced by the “strong growth” of microorganism cultures under the textile samples. While the fabrics covered with CR1 and CR2 were evaluated as having fungistatic effects against *C. albicans* cultures as well as bacteriostatic effects, the growth of microorganisms under the textile specimens was “moderate”, but quantitatively weaker on *S. aureus* cultures. By modifying the siloxane network, easier access of the dye to microorganisms can be facilitated. As the antimicrobial chromophore from the textile specimen diffuses into the environment, it can cause the stoppage of the multiplication of colonies, as in the case of CY22, which showed a bacteriostatic/fungistatic effect, or the death of the microorganism tested in the case of CR22 (bactericidal/fungicidal effect). Among the tested strains, the *S. aureus* strain was the most strongly inhibited, especially by CR12 and CR22, for which the count of viable cells on the textile samples was made after 24 h of contact of the textile samples with the inoculated microorganism. Assessment of the efficacy of the finished fabrics coated with CR12 showed the best results for dilutions of 10^−5^, CFU/mL = 3.6 × 10^8^, thus presenting a bacteriostatic effect determined by the presence of N,N-dimethylamine auxochrome groups characteristic of basic dyes recognized for these properties [14,55].

## 3. Conclusions

The multifunctional finishing of the surfaces of cotton fabrics with nanosols consisted in the embedding of two curcuminoid dyes in siloxane matrices, thus obtaining textile materials with fluorescent, antimicrobial, and hydrophobic properties.

The textile materials functionalized with fluorescent hybrids in the sol–gel system were obtained at low temperatures, with good dyeing yields and color stability. The dyeing processes were eco-friendly, bearing in mind that fluorescent hybrids are insoluble in water and are therefore easy to remove by filtration. By using yellow or red α,β-diketone derivatives in nanogel coating processes, functionalized textile materials with yellow-green or orange-red fluorescent properties were obtained. These materials can be used for signaling or in social media activities. Cotton textile materials covered with organic–inorganic composites depending on the nature of the host matrix and the auxochromes of the dye had registered contact angles between 129 and 145 degrees, thus showing hydrophobic properties and implicitly creating surfaces with self-cleaning capacity. The coatings on the cotton fabrics obtained by the sol–gel process showed good adhesion on the textile substrate, highlighted by very good properties in terms of resistance after fifteen repeated washing cycles and after being subjected to rubbing tests. By optimizing the structure of the siloxane network, it is possible to facilitate the exploitation of the antimicrobial properties of curcumin derivatives CY and CR and to obtain fabrics with an antimicrobial effect.

## 4. Materials and Methods

### 4.1. Materials

The curcuminoid dyes were synthesized and purified by a method previously published by us [50]. Compounds CY and CR were obtained at microwaves following the condensation reaction of the boric complex of acetylacetone with different benzaldehydes (4-hydroxy-in the case of CY and 4-N,N-dimethylamino- for CR). The aromatic aldehyde (7 mmol) and dodecylamine (0.162 mmol) were added over the boron complex (obtained by microwave irradiation at 300 W, 10 min of a mixture of 4 mmol, boron trioxide, 8 mmol, acetylacetone, and 3.2 mmol, tributyl borate). The mixture formed by the boric complex, aromatic aldehyde, and dodecylamine was irradiated at 100–500 W (depending on the chemical structure of the aromatic aldehyde) for about 20 min, after that, the mixture was cooled and added over a 10% acetic acid solution. The obtained suspension was filtered and the obtained solid was washed with distilled water until neutral pH, then purified by recrystallization from a mixture of ethyl acetate:methanol = 3:2 (*v*/*v*).

The following substances were used to obtain the nanosols in which the curcumin derivatives were hosted: tetraethylortosilicate (TEOS), dimethoxydimethylsilane (DMDMS), and dimethoxydiphenylsilane (DMDPS) from Aldrich, Saint Louis, MO, USA, with purity ≤95%, hydrochloric acid (0.1 N, HCl, Chimreactiv, Bucharest, Romania) ethanol (96%, EtOH, Chimreactiv, Bucharest, Romania), and tetrahydrofuran (99%, THF, Merck, Kenilworth, NJ, USA).

The fabric used in this study was 100% cotton with the characteristics of 79.4 ends, 65.4 picks, yarn count of 23.4 × 22 tex, and a weight of 161.98 g/m^2^, scoured and chemically bleached, obtained from Matasea Romana, Romania.

### 4.2. Methods

#### 4.2.1. Obtaining and the Method of Depositing Nanosols

Through the sol–gel process in the presence of TEOS as a generating agent and alkoxysilanes (with aliphatic or aromatic residues) as molecular precursors, following the hydrolysis and polycondensation reactions, the formation of the silica network takes place. These reactions can be directed according to the alkaline or acid environment in which they take place, thus generating particles with different structures and sizes. In our case, the hydrolysis and polycondensation reactions between TEOS and DMDMS, respectively DMDPS, took place in an acidic environment, thus favoring the hydrolysis reaction and obtaining film-forming materials. During the process of homo-condensation or hetero-condensation between TEOS and the network modifier, cyclic cages with different morphology are generated. The architecture of the silica network that is formed is directly influenced by the aliphatic or aromatic structure of the residues that the network modifier contains. The dyes are sequestered in these cages in the form of larger or smaller aggregates in relation to the topography of the host matrix [32,33]. By mixing the network initiator TEOS (1.6 mL) with the network modifying agent DMDMS or DMDPS (1.6 mL) in the presence of 0.1 N HCl (0.75 g) using a mixture of EtOH (2, 3 mL) and curcumin derivatives (0.02–0.06 g) dissolved in THF (2 mL) after 3 h under stirring, four types of nanocomposites were obtained. These differed in the type of silica network modifier, DMDMS (nanosol 1) or DMDPS (nanosol 2), and the curcumin derivative, bis-demethoxycurcumin (CY), 1,7-bis(4-N,N-dimethylphenyl)-1, 6-heptadiene-3,5-dione (CR), respectively. By loading only 0.02 g of dye into the siloxane matrix, coatings with low optical properties and no bacteriostatic effect against the tested microorganisms were obtained. Conversely, the introduction of a larger amount of dye (0.06 g) into the siloxane network, in order to obtain an antimicrobial effect as effective as possible on the textile fabrics, resulted in obtaining uneven colored coatings due to the aggregation processes between the dye molecules in the matrix. A total of 0.04 g of curcumin derivative was used for these nanocomposites, which is the optimal dye loading to obtain uniform coatings on cotton. The clear nanosol solutions with a content of 0.04 g of yellow or red dye was deposited on 2 g cotton fabric at a wet pick-up of 75 to 80% by the pad-drying process. The dyeing parameters of the cotton fabric were established on a horizontal laboratory padding mangle Ernst Bentz (Ernst Bentz AG, Kanton Zurich, Dielsdorf, Switzerland) at a constant speed of 0.5 m/min and a pressure of 0.4 kg/cm^2^. The cotton fabric was passed several times through the dyeing solution, and then dried at room temperature for 2 h. All cotton fabrics finished with nanosols were subjected to heat treatment at 120 °C for 1 h in a thermofixation oven, in order to complete the evaporation processes started at room temperature without affecting the morphological structure of the support fabric (Supplementary Materials, Figure S4).

#### 4.2.2. Characterization Methods

The following methods were used to characterize the structural and morphological properties of the coated textiles: FTIR-ATR spectra measurements of the nanosol coated fabrics were performed with a JASCO FT-IR 6300 instrument (Jasco Int. Co. Ltd., Tokyo, Japan) equipped with a Specac ATR Golden Gate (Specac Ltd., Orpington, UK) with KRS5 lens, in the range 400–4000 cm^−1^ (32 accumulations at a resolution of 4 cm^−1^). SEM images of the coated cotton samples were obtained with a scanning electron microscope (TM4000Plus; HITACHI, Tokyo, Japan) at an accelerating voltage of 10 kV and at magnifications up to 1800×. The porosity properties of the hybrid materials were evaluated by the BET (Brunauer–Emmett–Teller) and BJH (Barrett–Joyner–Halenda) methods, respectively. The specific surface areas (S_BET_) and total pore volumes (V_total_) of the samples degassed at 100 °C for 3 h were determined from the N_2_ adsorption–desorption isotherms measured at −196 °C using the Nova 2200e automated gas adsorption system Quantachrome (Quantachrome Instruments Corporate Drive, Boynton Beach, FL, USA). In order to establish the behavior of the textile materials finished with the hybrid nanosols at high temperatures, the thermogravimetric analysis of the samples was performed using a TGA Q5000IR instrument (TA Instruments, New Castle, DE, USA). The parameters of the thermogravimetric analysis for all samples were as follows: platinum vessels in which 2.5–3.2 mg of the sample were analyzed, using the method Hi-Res sensitivity 1.00, ramp 10 °C/min res 4 °C to 700 °C, synthetic air 5.0 (99.999%) was used as the purge gas at a flow rate of 50 mL/min. The surface topography of the coated surfaces was analyzed using a MultiMode 8 atomic force microscope (AFM) (Bruker, Santa Barbara, CA, USA). Measurements were carried out in Peak Force (PF) Quantitative Nanomechanical Mapping (QNM) mode, in air, at room temperature using silicon nitride tips at a scan rate of 1 Hz and a scan angle of 90°. NanoScope software (version 1.20, 2009, NanoScope Tech., Bedford, MA, USA) was used to process the data and images. The hydrophobic properties of the nanocomposite coated textiles were evaluated by performing six water contact angle (WCA) measurements on each sample using a CAM 200 (KSV Instruments, Helsinki, Finland) equipped with a high resolution camera (Basler A602f, Basler, Ahrensburg, Germany) and an autodispenser. WCA was measured in air, at room temperature, and ambient humidity for 6 µL drops of deionized water, distributed on the surface of each sample, after 2 s from impact. WCA was the average of the six measurements.

The evaluation of the optical properties of the nanosols deposited on the textiles was carried out by the following methods: the absorbance, diffuse reflectance spectra, and the total color differences in the CIELAB system using a 10° standard observer and illuminant D65 were measured with a V570 UV-VIS-NIR (Jasco Int. Co. Ltd., Tokyo, Japan) spectrophotometer equipped with a JASCO ILN-472 (150 mm) integrating sphere, using spectralon as the reference. The fluorescence steady state properties of the covered fabrics were analyzed by recording the fluorescence spectra on a JASCO FP 6500 spectrofluorimeter (Jasco Int. Co. Ltd., Tokyo, Japan), at 25 °C. Finished cotton fabrics were evaluated for color fastness to washing and rubbing using ISO 105-C06 [58] and ISO-105X12 [59], respectively. For these tests, 5 × 5 cm pieces of the four types of samples were used, which were each sewn between a piece of cotton and a piece of wool fabric. The obtained sandwiches were washed with an aqueous solution of 1% by weight sodium dodecyl sulfate at 60 °C for 1 h on a Linitest laboratory device (Atlas, Rock Hill, IL, USA). The samples were rinsed with hot and cold water, then opened and allowed to air dry. After washing and drying, finished cotton fabrics were tested according to standards for determining color change. The process was repeated, and after fifteen cycles of repeated washing tests, no significant change in the color of the evaluated samples was observed in relation to the original colored fabrics.

For the evaluation of antibacterial activity, the following strains of bacteria were used: *Staphylococcus aureus*, ATCC 25923 (*S. aureus*), *Escherichia coli*, ATCC 25922 (*E. coli*) and strain of fungi: *Candida albicans*, ATCC 10231 (*C. albicans*) from the Microbial Collection of ICECHIM. The evaluation of the antimicrobial activity was carried out by the diffusimetric disc method with spot inoculation (10 μL) for liquid samples and dyed fabric samples (2 × 2 cm) on the agar medium (Sabouraud medium for *C. albicans* and Muller Hinton medium for *E. coli* and *S. aureus*) inoculated into the cloth with the tested microorganisms. Antimicrobial activity was assessed by measuring the diameter of the clear area (halo) that appeared around the inoculation point or piece of tissue. The procedure of the antibacterial testing for the dyed fabric samples was based on a method described in ISO 20645:2004—“Textile fabrics—Determination of antibacterial activity—Agar diffusion plate test”. The working inoculum was represented by a suspension made from a fresh culture in AFS (sterile physiological water), developed on a solid TSA (Tryptic Soy Agar) medium, with a density of 1.5 × 10^8^ CFU/mL, nephelometrically adjusted (McFarland standard 0.5 = 1.5 × 10^8^ CFU/mL). The plates were incubated for 24 h at 28 °C for *C. albicans* and 37 °C for bacteria. Additionally, in the case of textile inoculation with *S. aureus* where the greatest inhibition (halo) was observed, the viable cells on the textile samples were counted after 24 h of contact of the textile sample with the inoculated microorganism. The textile sample was transferred into 20 mL of AFS and vortexed to help detach the bacterial cells from the textile support. The supernatant was serially diluted up to 10^−5^ dilution and 100 µL of each was inoculated on TSA medium. After 24 h of incubation, the colonies were counted. The number of viable bacteria was reported as colony forming units, CFU/mL.

## Figures and Tables

**Table 1:** Surface area, pore volume, and pore size of the coated cotton.

Sample	S_BET_ (m^2^·g^−1^)	V_tot_ (cm^3^·g^−1^)	D_BJH_ (nm)
Cotton	4.37	0.0056	2.97
CY1	2.28	0.0032	4.26
CY2	9.29	0.0091	3.45
CR1	3.58	0.0013	3.31
CR2	8.60	0.0050	2.99

**Table 2:** Absorbance at the maximum wavelength, color strength (K/S) of cotton textiles coated with nanosols and the evaluation of the films’ resistance during repeated washing cycles.

Sample	Abs. (λ_max_, nm)	K/S (a.u.)	K/S (a.u) Wash 1	K/S (a.u.) Wash 5	K/S (a.u.) Wash 15
CY1	0.7109 (422)	1.6668	1.5413	1.0610	1.0136
CY2	0.7462 (396)	1.8772	1.7476	1.0702	0.9453
CR1	0.7513 (440)	1.9087	1.4795	1.3469	1.2444
CR2	0.7694 (434)	2.0254	1.8306	1.3641	1.0893

**Table 3:** Evaluation of the washing fastness and rubbing fastness of the coating fabrics.

Sample	Washing Fastness (Grade) ISO 105 C06			Rubbing Fastness (Grade) ISO 105 X12	
	Color Change	Color Staining			
		Cotton	Wool	Dry	Wet
CY1	4–5	4–5	4–5	4	3
CY2	3	3–4	4	3–4	2–3
CR1	4–5	4	4–5	3–4	2–3
CR2	3–4	3–4	4	3–4	2–3

## Data Availability

The data are not publicly available due to containing information that could compromise the privacy of the research participants.

## Supplementary Materials

The following supporting information can be downloaded at: https://www.mdpi.com/article/10.3390/gels9050369/s1, Figure S1: Scheme for obtaining CY and CR dyes, encapsulated in siloxane matrices; Figure S2: Absorption spectra (a) and fluorescence spectra (b) of curcumin derivatives, in ethanol (conc. 1 × 10^−6^ mol/L); Figure S3: Color strength of cotton textiles coated and the films’ resistance during repeated washing cycles; Figure S4: The DSC measurements of fabrics finished with sol–gel nanocomposites; Table S1: Identification of dyes encapsulated in siloxane matrices.
