# Supplementary material for: Multifunctional Finishing of Cotton Fabric with Curcumin Derivatives Coatings Obtained by Sol–Gel Method
Source: Gels. 2023 Apr 30;9(5):369. doi: 10.3390/gels9050369 (PMC10217195; doi:10.3390/gels9050369)
Supplement: Supplementary file 1 [file gels-09-00369-s001.zip › gels-2347132-supplementary.pdf]

SUPLEMENTARY MATERIAL

# Multifunctional Finishing of Cotton Fabric with Curcumin Derivatives Coatings Obtained by Sol–Gel Method

Florentina Monica Raduly [1], Valentin Rădițoiu [1,*], Alina Rădițoiu [1], Adriana Nicoleta Frone [1], Cristian Andi Nicolae [1], Iuliana Răut [1], Mariana Constantin [1,2] and Maria Grapin [1]

The dyes CY and CR were obtained by Knoevenagel condensation, the reaction taking place through a nucleophilic addition of the methylene groups of acetylacetone to 4-hydroxybenzaldehyde or 4-N,N-dimethylaminobenzaldehyde, using the dodecylamineas an amino base catalyst. The structure of the two dyes differs in regard to the nature of the auxochrome groups grafted on the peripheral benzene nuclei of the diene chain of the 2,4-diketonic compounds. Thus, a curcumin derivative with hydroxyl groups (CY) and a derivative (CR) whose structure was grafted with N,N-dimethylamine auxochromes, were chosen for the study.

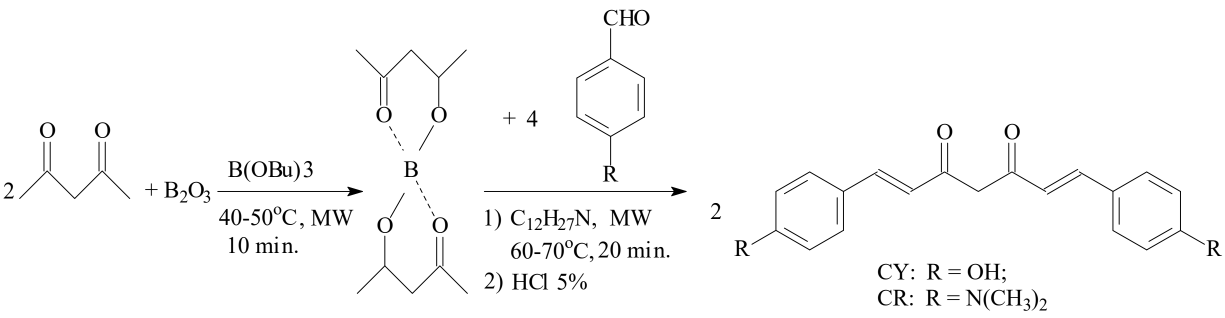


Figure S1. Scheme for obtaining CY and CR dyes, encapsulated in siloxane matrices.

Table S1. Identification of dyes encapsulated in siloxane matrices

| Dye | Molecular formula | Color | Yield [%] | Melting point [°C] |
|---|---|---|---|---|
| **CY** | $C_{19}H_{16}O_4$ | yellow | 78 | 176-177 |
| **CR** | $C_{23}H_{26}N_2O_2$ | red | 80 | 150-152 |

The structure of the molecules of curcuminoid dyes is mainly characterized by the π–π conjugated system of the heptadiene chain. This can be seen by analyzing the UV absorption spectra in which the transition corresponding $\pi \rightarrow \pi^*$ type from about 300 nm was a bathochromic shift ($\Delta \approx 115$ nm). The maximum absorption peaks were strongly influenced by auxochromes grafted onto the basic chemical structure of β–diketonic compounds. Compared to the CY compound, the CR derivative having an auxochrome containing a less electronegative atom (nitrogen), leads to a higher conjugation availability of unpaired electrons with aromatic residues. As a consequence, the intensity of the peak becomes lower, but

the band is larger and asymmetrical due to the appearance of a shoulder at about 500nm. However, regarding the fluorescence spectra, the intensities of the curcumin derivatives change, CY registering a decrease in the intensity of the fluorescence phenomena. The fluorescence quenching processes recorded in the case of CY compound is due to the intra- and intermolecular hydrogen bonds established between the hydroxyl auxochromes and the keto-enolic tautomers.

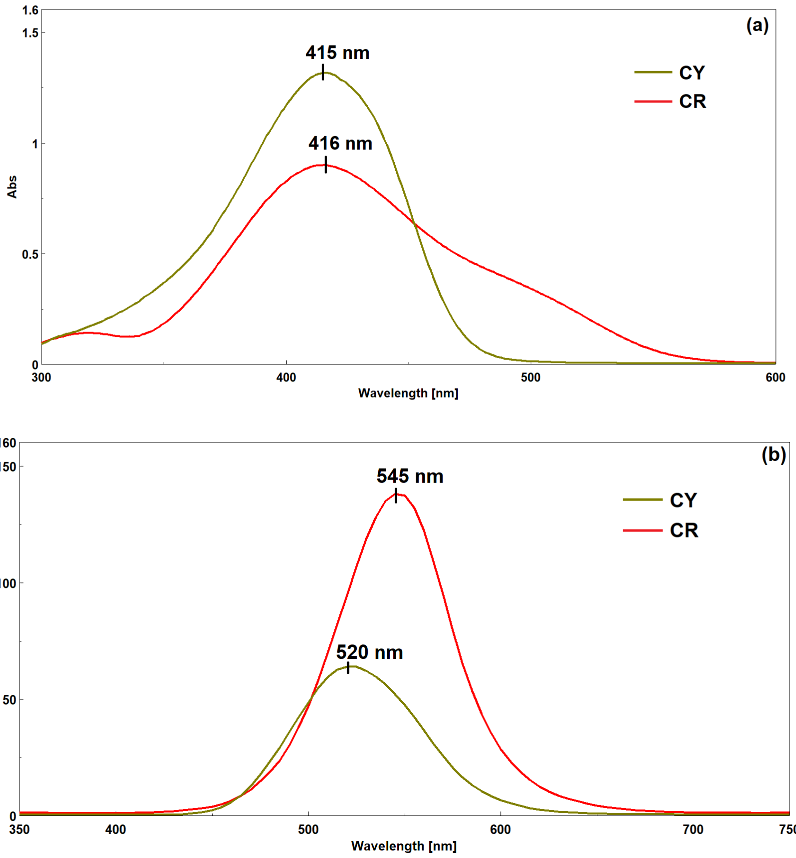


Figure S2. Absorption spectra (a) and fluorescence spectra (b) of curcumin derivatives, in ethanol (conc. $1 \cdot 10^{-6}$ mol/l).

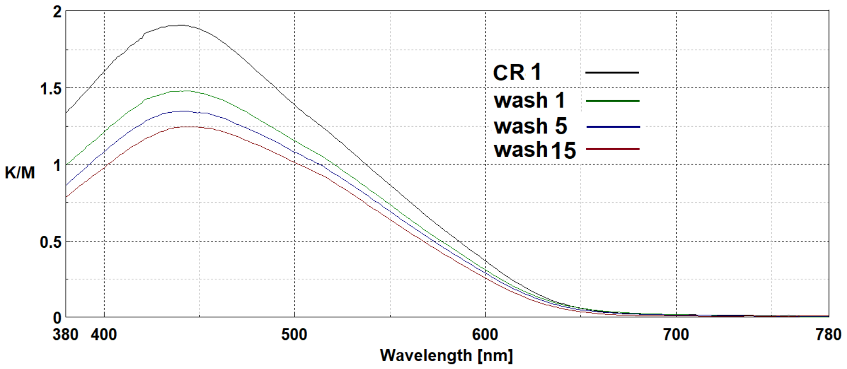


Figure S3. Color strength of cotton textiles coated and the films' resistance during repeated washing cycles.

An advantage of curcumin derivatives encapsulationin the siloxane matrices is the increasing quality of the finishes obtained on the fabrics. Therefore, the silica network through intermolecular interactions with the support fabrics leads to an increase of the rubbing fastness. By including the dye in the host matrices, homogeneous nanocomposites are obtained which do not preferentially separate after repeated washing processes as can be seen from the CR1 evaluation.

As can be seen from Figure S4, the DSC measurements for all samples subjected to heat treatment in the fixation oven show similar curves and values. From the data recorded for the thermal treatments to which the finished cotton fabrics were subjected, heating up to 120°C (a), isotherm for 60 min at 120°C (b), cooling to -75°C followed by a second heating up to 160°C (c), it can be seen that the samples were not affected during heat treatment at 120°C for 1 hour. All thermal events represent enthalpies of evaporation of volatile materials. On the isotherms recorded for the four types of fabrics finished with nanocomposites, no thermal events related to the phase transitions of the material such as the glass transition (Tg), melting or crystallization are highlighted.

The DSC measurements of the samples were performed using a DSC Q2000 instrument (TA Instruments, New Castle, DE, USA). The parameters at which the analysis was recorded for all samples are as follows: 3.00 mg (sample mass), pan: Tzero Aluminum, purge gas: Helium 5.0 (99.999%) 25 ml/min.

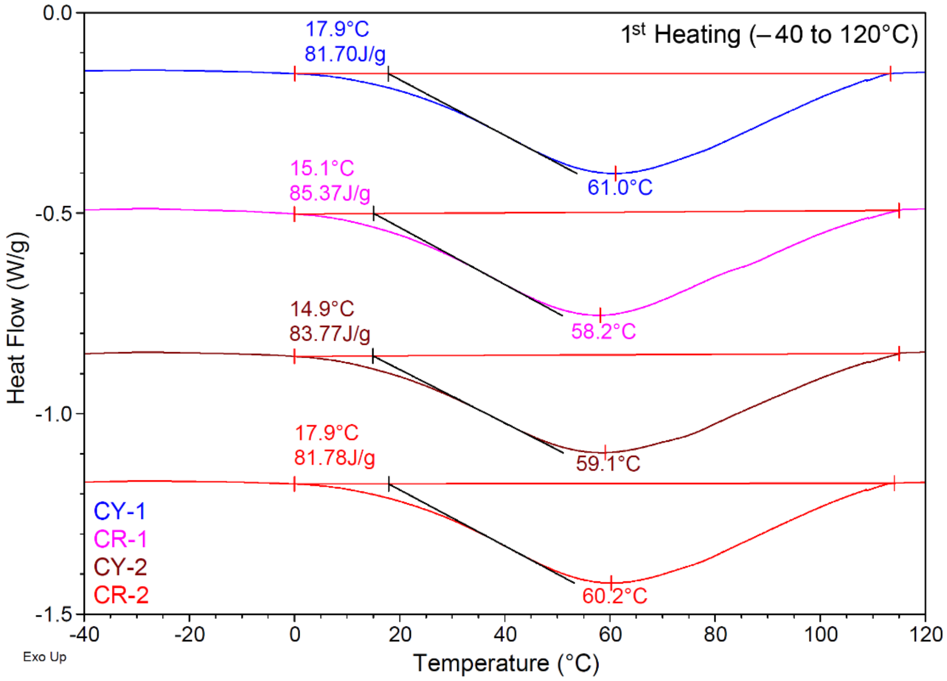


(a)

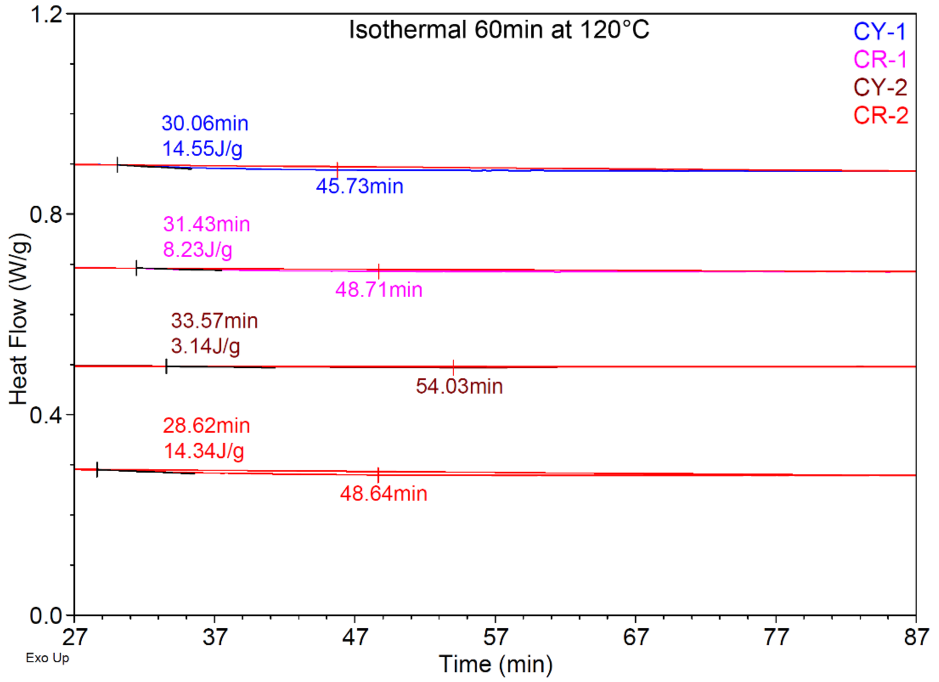


(b)

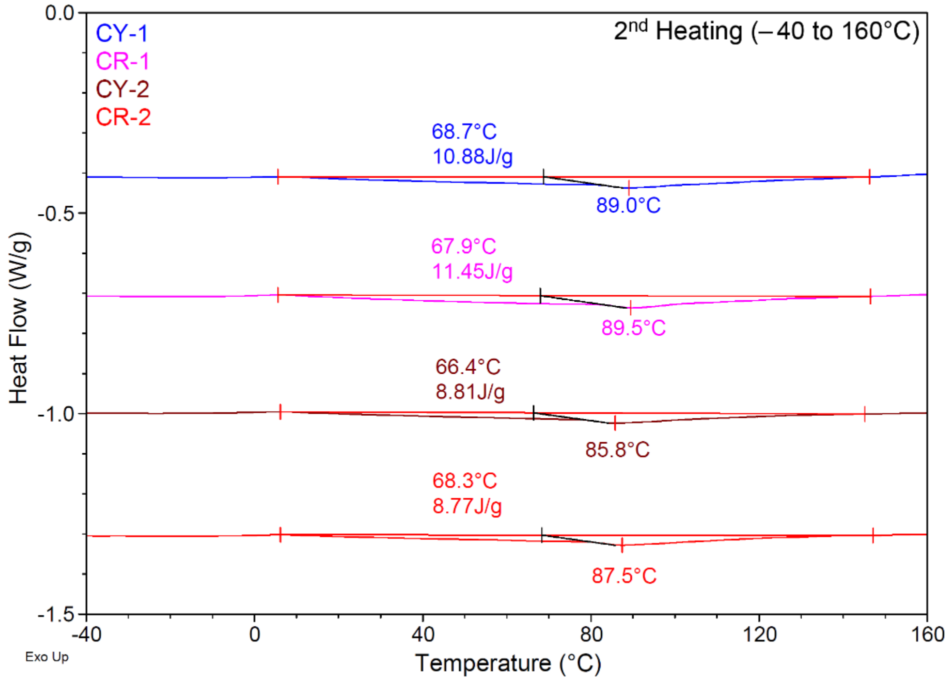


(c)

Figure S4. The DSC measurements of fabrics finished with sol-gel nanocomposites.
